# Developmental polychlorinated biphenyl exposure influences adult zebra finch reproductive behaviour

**DOI:** 10.1371/journal.pone.0230283

**Published:** 2020-03-19

**Authors:** Sara DeLeon, Michael S. Webster, Timothy J. DeVoogd, André A. Dhondt

**Affiliations:** 1 Department of Ecology and Evolutionary Biology, Cornell University, Ithaca, New York, United States of America; 2 Cornell Lab of Ornithology, Cornell University, Ithaca, New York, United States of America; 3 Department of Neurobiology and Behaviour, Cornell University, Ithaca, New York, United States of America; 4 Department of Psychology, Cornell University, Ithaca, New York, United States of America; Claremont Colleges, UNITED STATES

## Abstract

Polychlorinated biphenyls (PCBs) are worldwide chemical pollutants that have been linked to disrupted reproduction and altered sexual behaviour in many organisms. However, the effect of developmental PCB-exposure on adult passerine reproductive behaviour remains unknown. A commercial PCB mixture (Aroclor 1242) or an estrogenic congener (PCB 52) were administered in sublethal amounts to nestling zebra finches (*Taeniopygia guttata*) in the laboratory to identify effects of developmental PCB-exposure on adult zebra finch reproductive parameters. Results indicate that although traditional measures of reproductive success are not altered by this PCB dosage, PCBs do alter sexual behaviours such as male song and nesting behaviour. Males treated with PCB 52 in the nest sang significantly fewer syllables than control males, while females treated with Aroclor 1242 in the nest showed the strongest song preferences. PCB treatment also caused an increase in the number of nesting attempts and abandoned nests in the Aroclor 1242 treatment relative to the PCB 52 treatment, and offspring with control fathers fledged significantly earlier than those with fathers treated with Aroclor 1242. Behavioural differences between males seem to best explain these reproductive effects, most notably aggression. These findings suggest that sublethal PCB-exposure during development can significantly alter key reproductive characteristics of adult zebra finches, likely reducing fitness in the wild.

## Introduction

Polychlorinated biphenyls (PCBs) are a class of syththetic chemicals that are ubiquitous in the environment. They were produced commercially for use in industrial products, such as dielectric, hydraulic and heat transfer fluids [1]. PCBs have a di-benzene backbone, one to ten degrees of chlorination and were manufactured in mixtures of structurally related compounds known as congeners [2]. Congener mixture production was banned in the United States in the late 1970s, however, the chemical mixtures are still present and identifiable in the environment [3]. There are 209 different PCB congeners with different modes of action and complex individual and interactive effects [4]. Exposure to high levels of most PCB congeners can be lethal and highly chlorinated PCBs are more toxic than PCBs with fewer chlorine substitutions [5]. Additionally, high-level exposure to PCBs with fewer chlorine substitutions causes endocrine disruption [6,7], while low-level PCB exposure has sublethal effects. Due to similarities between the chemical structure of PCBs and sex steroids, sublethal PCB-exposure may cause behavioural changes, especially affecting reproductive behaviours that are regulated by hormones [8–10].

While organisms in the wild can experience long-term or chronic PCB exposure [11–13], captive studies show that the timing of PCB exposure is particulary influential. Organisms may be especially sensitive to PCB exposure during development (e.g. [14]), with lasting consequences into adulthood [15]. Indeed, the developmental stress hypothesis postulates that adult morphology and behaviour is of evolutionary significance because it indicates whether an individual received adequate nutrition during development [16–18]. Decades of studies indicate that PCB exposure during development can cause long-lasting effects that are influenced by the degree and location of congener chlorination. For example, when pregnant female rats are treated with the weakly estrogenic PCB mixture Aroclor 1221, their offspring show decreased anxiety behaviour, while exploration increases in male, but not female, offspring [19].

In birds, PCB exposure is also correlated with disrupted reproductive behaviours. A field study of great black-backed gulls (*Larus marinus*) shows that females exposed to PCBs through their food source have higher PCB blood concentrations, correlating with a decline in egg laying [20]. Other field studies show that in female tree swallows (*Tachycineta bicolor*), PCB exposure through food is linked with changes to plumage, clutch size, and nest building behaviour [21–23]. In captivity, American kestrels (*Falco sparverius*) fed with PCB-contaminated food exhibit altered mating, nesting and incubation behaviour, as well as altered chick development [24–26]. Additionally, feeding PCBs to adult female zebra finches (*Taeniopygia guttata*) affects incubation time, the number of nests built and the number of clutches laid [27].

Despite many studies investigating the consequences of PCB exposure, we know of no studies in passerines that isolate the effects of nestling PCB exposure to the reproductive characteristics and behaviours of the adult. Understanding exposure during the nestling stage is critical because in polluted areas it is likely adult passerines are feeding contaminated insects to their offspring [28–30], exposing their offspring to potential long-term impacts of PCBs on development and adult behaviours. Subsequent effects are likely to affect populations well beyond locations of contamination because natal dispersal in males moves them from their nestling location [31].

This study makes an important contribution to the currently limited experimental evaluation of the effects of nestling PCB exposure on adult reproductive characteristics and behaviour in zebra finches. We focus on two types of PCBs, selected to mimic environmental exposure and estrogenic affects, and administer them at sublethal doses during the nestling stage to determine impacts on reproductive behaviour once individuals reach adulthood. We measure key reproductive endpoints that influence the success of breeding, including male song, female song preference, male reproductive behaviour, and male brain anatomy in key locations within the brain. These varied endpoints allow for a more comprehensive assessment of the consequences of developmental PCBs on reproductive characteristics in the adult bird. Furthermore, these results could help explain the correlations found between PCB load and behavioural variations in wild birds present in areas of PCB contamination [32].

## Materials and methods

### Experimental subjects

All zebra finches used in this study were reared and housed at Cornell University (Ithaca, New York USA) using protocols approved by the Cornell University Institutional Animal Care and Use Committee (IACUC Protocol 1988–0135). All subjects were fed Kaytee^™^ forti-finch diet (Kaytee Products, Inc., Chilton, Wisconsin USA), oyster shell, cuttlebone, and water *ad libitum*. Subjects were housed in rooms on a 14L: 10D photoperiod, with 40–50% relative humidity, and an average temperature of 22°C.

To obtain the subjects for this study, thirteen healthy zebra finch pairs (F0) with prior successful breeding experience were housed as pairs in 13 breeding cages (61.0cm x 35.6cm x 40.6cm) equipped with one plastic nest cavity and coconut fiber nesting material. F1 offspring were individually identified by daily marking each nestling with coloured Crayola^™^ non-toxic permanent markers until day 14 when they were uniquely colour banded for identification (Avinet, Inc. Dryden, New York USA). Individual males and females (F1) were identified based on genetic sex determination [33,34] (see Supporting information for details), and randomly assigned to a treatment. Sex was confirmed with adult plumage by day 60.

### Experimental design

**Fig 1:**
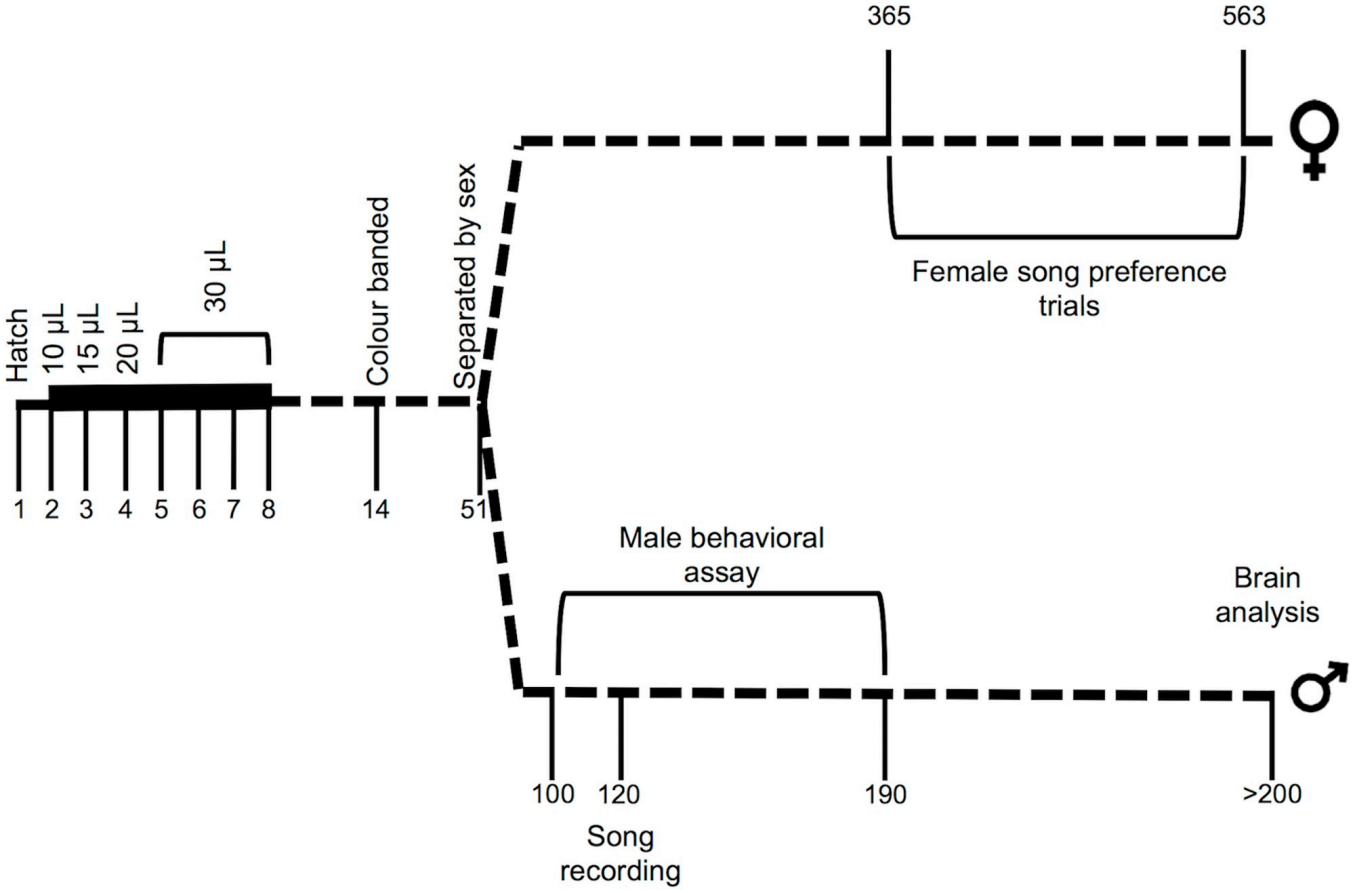
Experimental timeline of F1 male and female zebra finches. Unit-less numbers on the timeline correspond to days.

We randomly assigned nestlings (F1) to a PCB treatment, the commercial mixture Aroclor 1242 or the estrogenic congener PCB 52, or a control group (see Supporting information for further details). Nestlings assigned to the Aroclor 1242 or PCB 52 groups were administered a total of 165μL of PCB (AccuStandard.com) dissolved at 1-mg/mL in Canola oil, while control nestlings received 165μL of only Canola oil [35] (Fig 1). The treatments were administered orally across seven days during the most influential period for exogenous estrogen effects on sexual differentiation [36]:10μL on day 2, 15μL on day 3, 20μL on day 4, and 30μL on days 5–8. PCB dosage was determined based on the range of exposure nestlings are likely to encounter in areas of environmental PCB contamination [29,37–40].

Fledglings remained in their natal cage with their parents until post-hatch day 50, which is approximately the middle of their sensitive song-learning phase [41]. From hatching until day 50, F1 males were not in auditory isolation from their father or neighboring males. On post-hatch day 51 fledglings were placed in single-sex aviaries (91.4cm x 61.0cm x 122.0cm) in a room separate from their parents, but not in auditory isolation from other zebra finch adult males.

### Male song recording

F1 males (Control: *N* = 17; Aroclor 1242 and PCB 52: *N* = 7 for each) were individually recorded for one hour in a soundproof room on day 120±1, when adult male zebra finch song is stable [42] (Fig 1). Males were placed in the soundproof room at least one hour prior to the recording to acclimate. A single, untreated, unrelated adult female from the colony was placed in a separate cage and used as a stimulus in all song recordings. Males were recorded for up to three days, as needed, to obtain at least 10 song recordings [43,44].

Spectrograms generated in RavenPro 1.4 (Bioacoustics Research Program, Cornell University, Ithaca, New York USA), were used to measure song motif length, peak frequency, syllable number (less complex songs having fewer syllables [44]), and song rate (syllable number/motif length). Sound Analysis Pro (SAP) was used to measure similarity, accuracy, and sequence within a male’s own song (see Supporting information for further details).

### Male behavioural assay

When the F1 males were a minimum of 100 days post-hatch (Fig 1) they were tested for the effectiveness of their courtship and parental behaviors. Thirteen groups consisting of three zebra finches each were placed in experimental cages: 1) one F1 male from the control group, 2) one F1 male from either the Aroclor 1242 group (*N* = 7) or the PCB 52 group (*N* = 6), and 3) one inexperienced and unrelated female from the colony with no PCB-exposure (see Supporting information for further details). Additionally, prior to the grouping the colour bands placed on the F1 males as nestlings were removed. The males were given a single black leg band uniquely placed to identify each male and to prevent any preference for band colour from the female [45]. For simplicity, ‘PCB treatment’ terminology in the results refers to the experimental male in the cage.

Prior to the beginning of the behavioural assay, weight and tarsus length were measured in the females and F1 males. When possible, the Aroclor 1242- and the PCB 52- treatments contained male pairs that were matched by family (i.e. male siblings were preferentially paired), size (weight and tarsus length), and age.

### Behavioural observations

Ten-minute observation periods of the triad of zebra finches in the male behavioural assay began on the second day of the experiment. The observation periods occurred on every second day in a randomized order, in the mornings, within the first three hours of lights on, when the birds were most active. Observations continued daily for 90 days or until 14 days after the last fledgling event of the first successful clutch, whichever first occurred. The first successful clutch was defined as the first clutch within the 90-day period that fledged.

Behavioural observations were grouped into three classifications: pre-laying (before the first egg was laid), laying (after the first egg was laid, but before the first egg hatched, which includes the incubation period), and post-hatch (after the first egg hatched). During the pre-laying and laying period the number of one-minute intervals in which courtship behaviours (male singing, allopreening, and time in the nest box with the female) were observed at least once were recorded. During the post-hatch period the number of one-minute intervals in which courtship and parenting behaviours (male singing, allopreening, time in the nest box with the female, food provision to hatchlings) were observed at least once were recorded. During the pre-laying and laying period the number of ten-minute observation periods in which aggressive behaviour (i.e. chasing another individual) was observed at least once were recorded. In addition, the nest boxes in each cage were monitored and nesting and reproductive characteristics (e.g. latency to nest building, number of nests built, latency to laying, numbers of eggs laid before a successful clutch, number of clutches, clutch size, mortality, fledgling age, fledgling size, etc.) were measured. Offspring from this behavioural assay with F1 fathers are defined as F2 offspring.

### Genetic parentage

F2 hatchling parentage was genetically determined using microsatellites evaluated using pinfeathers and tissue (see Supporting information for details). Between two and five pinfeathers were collected from living F2 fledglings and tissue samples were taken from F2 nestlings that died in the nest. The quills of the feather samples were immediately submerged in buffer solution and stored at room temperature. Tissue samples were frozen at -20°C immediately after collection until analysis.

### Female song preference trials

Proximity (or association) song preference trials for adult F1 female subjects (Control: *N* = 7; Aroclor 1242: *N* = 6; PCB 52: *N* = 7) began when females were approximately 365 days old and continued for about six months (see Fig 1). Song preference trials were performed following protocol by Lauay et al. [46] (see Supporting information for details), performed in the morning between 0700–1100, and the investigator was blind to the experimental treatment of the female during data collection.

#### Stimulus recordings

Females were exposed to three types of song choice trials, where each trial consisted of two recorded males singing where the female could make a choice. The first trial type was a choice between archived recordings of songs from two untreated males, one raised with a tutor, or ‘social’ (*N* = 4) and one without a tutor, or ‘isolate’ (*N* = 4). Untreated female zebra finches had been previously shown to be able to distinguish between these two song treatments with this experimental set-up [46]. The second and third trial types were recordings from experimental F1 males, where the second trial type was a choice between the songs from control males (*N* = 4) and Aroclor 1242-treated males (*N* = 4), and the third between the songs from control males (*N* = 4) and PCB 52-treated males (*N* = 4). Each female underwent seven trials: one with the ‘social’ vs. ‘isolate’ song types and three trials each with the ‘Control’ vs. ‘Aroclor 1242’ song types and ‘Control’ vs. ‘PCB 52’ song types (see Supporting information for details). No female was presented with the song of a family member. If there were male siblings from different treatment groups, their songs were paired in the trials.

### Male histology and dendritic spine quantification

We extracted the whole brain for each male between day 200 and 300 after males completed the behavioural assay (Fig 1). Birds were anesthetized with 0.1 mL Chloropent and transcardially perfused with 0.9% saline followed by 10% formalin. Immediately after extraction, the fresh brain was weighed (Control: *N* = 14; Aroclor 1242: *N* = 6; PCB 52: *N* = 6) and then immersed in Golgi-Cox solution [47] for approximately 6 weeks, with the solution changed after the first week, when the brain was weighed again. The tissue was dehydrated and embedded in celloiden, sectioned at 100μm in the coronal plane, reacted with ammonia, counter-stained with methylene blue and cresyl violet, and mounted on slides. Testes were also removed and weighed (Control: *N* = 14; Aroclor 1242: *N* = 7; PCB 52: *N* = 7).

Dendritic spines were quantified in the HVC, RA, and hippocampus. Two human judges blind to the experimental treatment correlated 0.99 for the hippocampus quantification, and a single observer, also blind to the experimental treatment, quantified all data from the HVC and RA. In all dendritic spine quantification, neurons from a brain region were randomly and blindly chosen by scanning the regions for fully stained neurons with dendrites parallel to the sectioning plane. Within each of the nuclei regions, the number of spines was counted on three dendrites using 945x magnification with oil. Three 11–12μm sections along each of the three dendrites were quantified: a proximal location (approximately 11–12μm from the soma), a medial location (approximately 22–24μm from the soma), and a distal location (the last 11–12μm, usually approximately 40μm from the soma). When possible, all three locations were measured from the same dendrite. When it was not possible to get all measurements from the same dendrite, additional measurements were taken from other dendrites that were parallel to the sectioning plane and fully stained to increase the sample size. Spines were counted on different regions of the dendrite to control for environmental and developmental variations in spine count and position [48,49]. All spines were counted, including those that looked like a swelling of the dendritic surface, and spines that were long, thin projections (see [50] for photographs of spine variation). Final sample size for the dendritic quantification was *N* = 11 (Control: *N* = 3; Aroclor 1242: *N* = 5; PCB 52: *N* = 3) for the HVC, *N* = 14 (Control: *N* = 6; Aroclor 1242: *N* = 6; PCB 52: *N* = 2) for the RA, and *N* = 16 (Control: *N* = 5; Aroclor 1242: *N* = 6; PCB 52: *N* = 5) for the hippocampus.

### Statistical analysis

All variables were first tested for normality and homogeniety of variance with a Shapiro-Wilk test and Levene’s test, respectively. To satisfy parameters of normality specified variables were transformed with a Box Cox transformation. If the distribution of a variable deviated from normality despite transformation, analyses were performed with non-parametric statistical tests. All other analyses were performed with parametric statistics. A *P*-value of <0.05 was required for significance. All analyses were performed with JMP^®^ 9 (SAS Campus Drive, Cary, North Carolina USA).

Song characteristics were analyzed with one-way ANOVAs (general linear models) with the experimental treatment as a fixed effect and the genetic family of the individual as a random effect.

Measurements prior to the start of the behavioural assay from F1 males and females, as well as F2 measurements, were analyzed with one-way ANOVAs (general linear models) with the experimental treatment as a fixed effect and the genetic family of the individual as a random effect. Measurements of reproductive success of the F1 males were analyzed with one-way ANOVAs (general linear models) with the genetic father as a fixed effect and the behavioural assay of the father as a random effect.

If courtship behaviours occurred at any time during the one-minute interval, the bird was scored as performing that behaviour during that minute, for a score of one. For aggressive behaviours, each ten-minute observation period was considered the unit of measurement. If an aggressive behaviour occurred at any time during the ten-minute observation period, the bird was scored as performing that behaviour during that observation period, for a score of one. The daily scores for courtship and aggressive behaviour were summed for each F1 male during each behavioural period (pre-laying, laying, post-hatch) and analysed as count data. Statistical differences were determined using a T-test or a Kruskal-Wallis test.

In cages where an adult male died or the behavioural assay was discontinued due to safety reasons, the behavioural stage during which the assay was stopped is excluded from the behavioural analysis, and only cage treatments with complete prior behavioural stages are included. Nesting and reproductive characteristics were analysed using a T-test or a Kruskal-Wallis test.

A mixed model was used to test for effects of treatment on female size (tarsus length and body weight at day 20 and day 120) and age at fledging, with treatment and mean tarsus length of the parents as fixed effects, and family and observer as a random effect.

Female location in the experimental set-up was recorded at the beginning of every 15-second interval, for a total of 33 measurements for each of the two 8-minute observation periods (track 1 and track 2) for a maximum possible count of 66 measurements. Females that did not move to an end region (S1 Fig) during the duration of the trial were removed from the analysis. The location of the female in the end regions (S1 Fig) was interpreted as a preference for song coming from the closest speaker and results are reported as the average number of counts that a female made a choice. Because sublethal levels of PCBs have shown to affect bird song [32], there were *a priori* reasons to expect differences between the groups. Therefore, paired T-tests were performed to analyse female preference for song.

Total brain mass and testes mass were analyzed with one-way ANOVAs where treatment was a fixed effect and age at perfusion was a random effect. Due to small sample sizes, the number of dendritic spines were analyzed with Student’s T-tests.

## Results

### Male song characteristics

**Fig 2:**
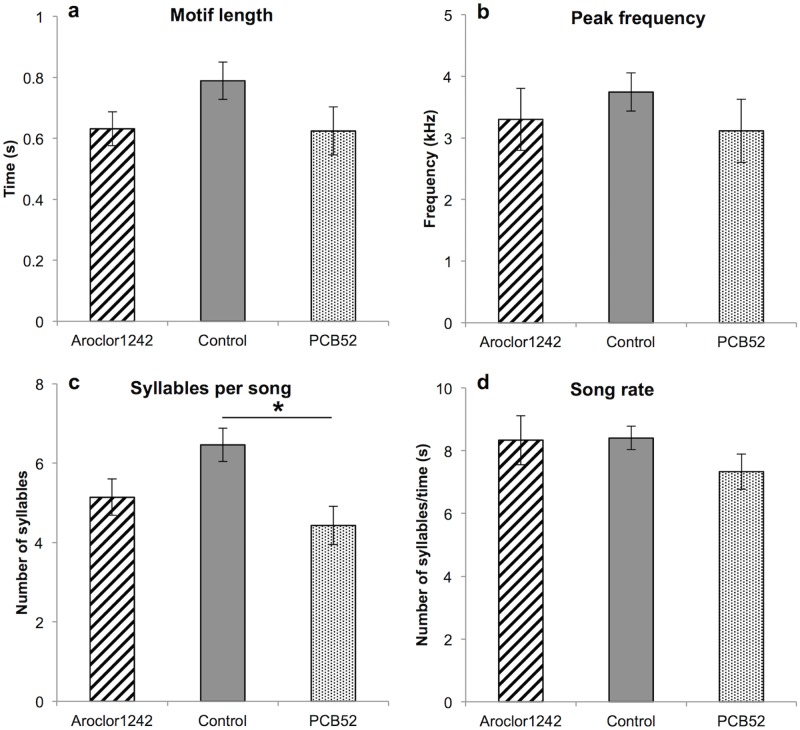
Adult male song characteristics are influenced by PCB exposure in the nest. The mean value of adult song characteristics (±SE) of developmentally treated Aroclor 1242, control, and PCB 52 male zebra finches. Significant differences are indicated with an asterisk (*).

Song motif length, peak frequency within the motif, and song rate did not differ among male zebra finches in the three treatment groups (Song motifs: *F*_2,28_ = 1.79, *P* = 0.19; Peak frequency: *F*_2,28_ = 0.69, *P* = 0.51; Song rate: *F*_2,28_ = 1.62, *P* = 0.22; Fig 2). However, the males in the three groups differed in the number of syllables within their song motifs (*F*_2,28_ = 4.08, *P* = 0.03; Fig 2c), where the control group sang significantly more syllables in their song motifs than individuals treated with PCB 52. No differences in any measurements of song consistency within an individuals’ own song were identified (Similarity: *F*_2,28_ = 2.54, *P* = 0.10; Accuracy: *F*_2,28_ = 2.71, *P* = 0.08; Sequence: Kruskal-Wallis: *H*_*3*_ = 0.29, *P* = 0.87).

### Male behavioural assay

There were no differences between F1 male treatment groups in fledgling age, D20 body mass or tarsus length, D120 body mass or tarsus length, body mass at experiment start, or tarsus length at experiment start (S1 and S2 Tables). There was also no difference in body mass or tarsus length at experiment start between the adult females in the two types of treatment cages (S2 Table).

The assay was stopped in four cages due to extreme and continuous aggression between adult males. In one Aroclor 1242 treatment cage the assay was stopped (laying stage) due to the aggression from the control male towards the Aroclor 1242 male. The assay was also stopped in two separate PCB 52 treatment cages (pre-laying stage) due to the aggression from the control male towards the PCB 52 male. In one PCB 52 treatment cage the assay was stopped (post-fledgling stage) due to aggression from the PCB 52 male towards the control male. Additionally, the assay was stopped in one Aroclor 1242 treatment cage (post-hatch period) and in one PCB 52 treatment cage (laying stage) due to the death of the respective control males from unknown causes. However, there were no differences in the levels of measured aggressive behaviours in the pre-laying or laying period in either experimental treatment (S3 Table).

#### Reproductive behaviour

With the exception of singing, reproductive behaviours did not differ between the control and treatment males, within the treatments or between the treatments (S4 Table) in the pre-laying, laying or post-hatch periods. During the pre-laying period, control males in the Aroclor 1242 treatment cages sang significantly more than control males in the PCB 52 treatment cages (Kruskal-Wallis: *H*_*2*_ = 4.40; *P* = 0.04), although the amount of total singing in the pre-laying period did not differ between treatment cages (Student’s T-test: *t*_*9*_ = -2.19, *P* = 0.06). During the laying period the amount of total singing in the Aroclor 1242 treatment cages was greater than the amount of total singing in the PCB 52 treatment cages (Student’s T-test: *t*_*7*_ = -3.60, *P* = 0.01).

#### Reproductive characteristics

There is no evidence that the experimental treatment to the F1 nestling males compromised their fertility as adults, individuals from each treatment group sired genetic offspring. There was only one case of mixed paternity in the behavioural assay, where both the control male and the PCB 52- treated male in one cage each sired offspring. In all other cages only one male sired the entirety of the offspring from that cage.

The two cage treatments did not differ in the latency to nest building, laying, or hatching; number of eggs hatched; number of eggs per clutch; total number of eggs; or number of eggs abandoned (Table 1). There were no differences in any traditional measures of reproductive success between the F1 PCB-treated and control males (Table 1). The Aroclor 1242 cage treatment built significantly more nests before a successful clutch than the PCB 52 cage treatment (Table 1). Females in the Aroclor 1242 cages laid a significantly greater number of eggs before their first successful clutch than the females in the PCB 52 cages (Table 1). The PCB 52 cage treatment also had significantly earlier successful clutches than the Aroclor 1242 cage treatment (Table 1).

**Table 1:** Male behavioural assay nesting characteristics. Significant differences are indicated with an asterisk (*) and darker shaded cells.

	Aroclor 1242 cage treatment	PCB 52 cage treatment	Statistics	*P*
Nest building latency (days)	2.57±0.97 (*7*)^a^	7.00±3.53 (*6*)	Kruskal-Wallis: H(2) = 1.35	0.25
# of nests built	6.57±1.34 (*7*)	2.00±0.00 (*6*)	Kruskal-Wallis: H(2) = 7.87	0.005*
Laying latency (days)	14.71±3.24 (*7*)	11.33±3.41 (*6*)	Kruskal-Wallis: H(2) = 0.52	0.47
# of first hatching clutch	2.20±0.32 (*7*)	1.20±0.18 (*6*)	Student’s T-test: t(11) = -2.36	0.03*
# eggs accumulated until first successful clutch	7.20±1.68 (*7*)	1.60±1.25 (*6*)	Student’s T-test: t(11) = -2.33	0.03*
Hatching latency (days)	54.60±5.99 (*5*)	35.20±10.46 (*5*)	Student’s t-test: t(8) = -1.61	0.15
# eggs hatched	3.29±1.32 (*7*)	2.17±0.60 (*6*)	Kruskal-Wallis: H(2) = 0.13	0.72
# eggs/clutch	4.00±1.07 (*7*)	3.83±0.87 (*6*)	Kruskal-Wallis: H(2) = 0.27	0.60
Total # of eggs	20.14±2.71 (*7*)	18.00±1.93 (*6*)	Student’s T-test: t(11) = -0.64	0.53
# of eggs abandoned	4.57±1.84 (*7*)	4.67±2.70 (*6*)	Kruskal-Wallis: H(2) = 0.02	0.89
Hatching success (# of eggs hatched/clutch size)	0.57±0.10 (*7*)	0.56±0.18 (*6*)	Student’s T-test: t(11) = -0.04	0.97
Fledging success (# of eggs fledged/clutch size)	0.40±0.16 (*7*)	0.56±0.09 (*6*)	Student’s T-test: t(11) = 0.75	0.76

^**a**^All values are mean±SE (*N*)

The F2 fledgling age was significantly different between the F1 genetic fathers (Table 2). A Tukey’s post hoc test shows that the F2 offspring in the control group fledged significanlty earlier than offspring with genetic fathers treated with Aroclor 1242, but not PCB 52, which had intermediate fledling age (Table 2). There were no significant differences between the number of F2 offspring hatched, fledged, and the mass or tarsus length in the F2 fledglings based on their genetic parentage (Table 2). There was also no difference between the number of male or female F2 offspring from the F1 genetic fathers (Table 2).

**Table 2:** Male behavioural assay offspring (F2) characteristics. Significant differences are indicated with an asterisk (*) and darker shaded cells.

		Control	Aroclor 1242	PCB 52	*F*	df	*P*
F2 offspring hatched		1.40±0.54 (*10*)^a^	3.40±1.89 (*5*)	1.00±0.55 (*5*)	Kruskal-Wallis: H(3) = 0.80		0.67
F2 offspring fledged		1.20±0.55 (*10*)	2.20±1.02 (*5*)	1.00±0.55 (*5*)	Kruskal-Wallis: H(3) = 0.89		0.64
F2 offspring fledgling age (days)		18.67±0.57 (*12*)	20.91±0.56 (*11*)	19.20±0.38 (*5*)	4.67	25	0.02*
F2 offspring fledgling mass (g)		11.00±0.20 (*12*)	11.27±0.48 (*11*)	10.90±0.25 (*5*)	0.25	25	0.57
F2 offspring tarsus length (mm)		14.12±0.18 (*12*)	13.93±0.16 (*11*)	13.83±0.19 (*5*)	0.57	25	0.57
F2 sex ratio	M	1.50±0.65 (*4*)	1.67±0.88 (*3*)	0.33±0.33 (*3*)	0.60	7	0.58
	F	1.50±0.65 (*4*)	1.67±0.33 (*3*)	0.33±0.33 (*3*)	0.04	7	0.96

^**a**^All values are mean±SE (*N*)

### Female song preference assay

**Fig 3:**
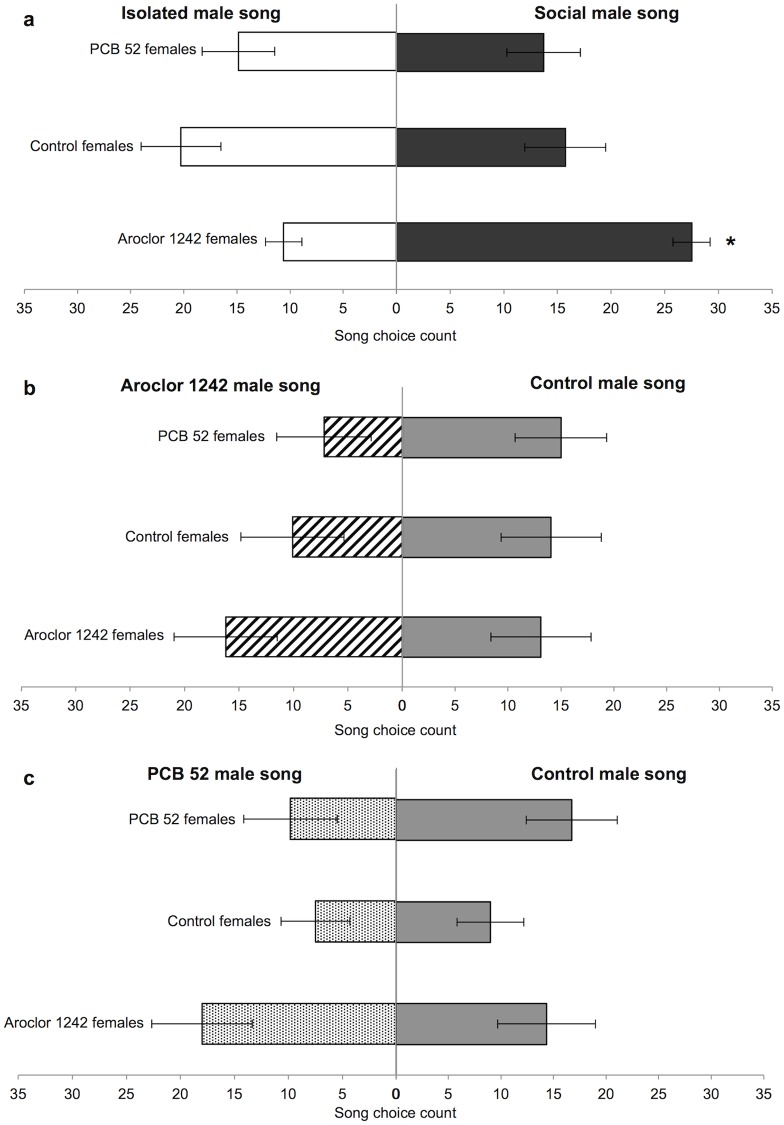
F1 female song preference assay. The average number of counts that a female made a choice between a) social/isolate male songs, b) control/Aroclor 1242 male songs, and c) control/PCB 52 male songs. Bars are mean±SE; significant differences are indicated with an asterisk (*).

All measurements of F1 female fledgling age and size (day 20 weight and tarsus length, day 120 weight and tarsus length) were not different between treatment groups (S1 Table). Out of the total 265 trials, females made a choice in 228 (86%). When the three female groups were presented with social male song in comparison to isolated male song, only females treated with Aroclor 1242 significantly preferred the social male song (Paired T-test: *P* = 0.004, Fig 3a). When the three female groups were presented with the control/Aroclor 1242 male song-choice they showed no preference for either song type (Paired T-test: *P*>0.05, Fig 3b). Similarly, the three female groups also showed no preference when presented with the control/PCB 52 male song-choice (Paired T-test: *P*>0.05, Fig 3c).

### Male histology and dendritic spine quantification

There were no differences between the three treatment groups in total brain mass immediately after perfusion or after the brain was immersed in Golgi-cox solution for one week (S5 Table). Each male in this experiment had two testes and there was no difference between the three treatment groups in testes mass (S5 Table).

**Fig 4:**
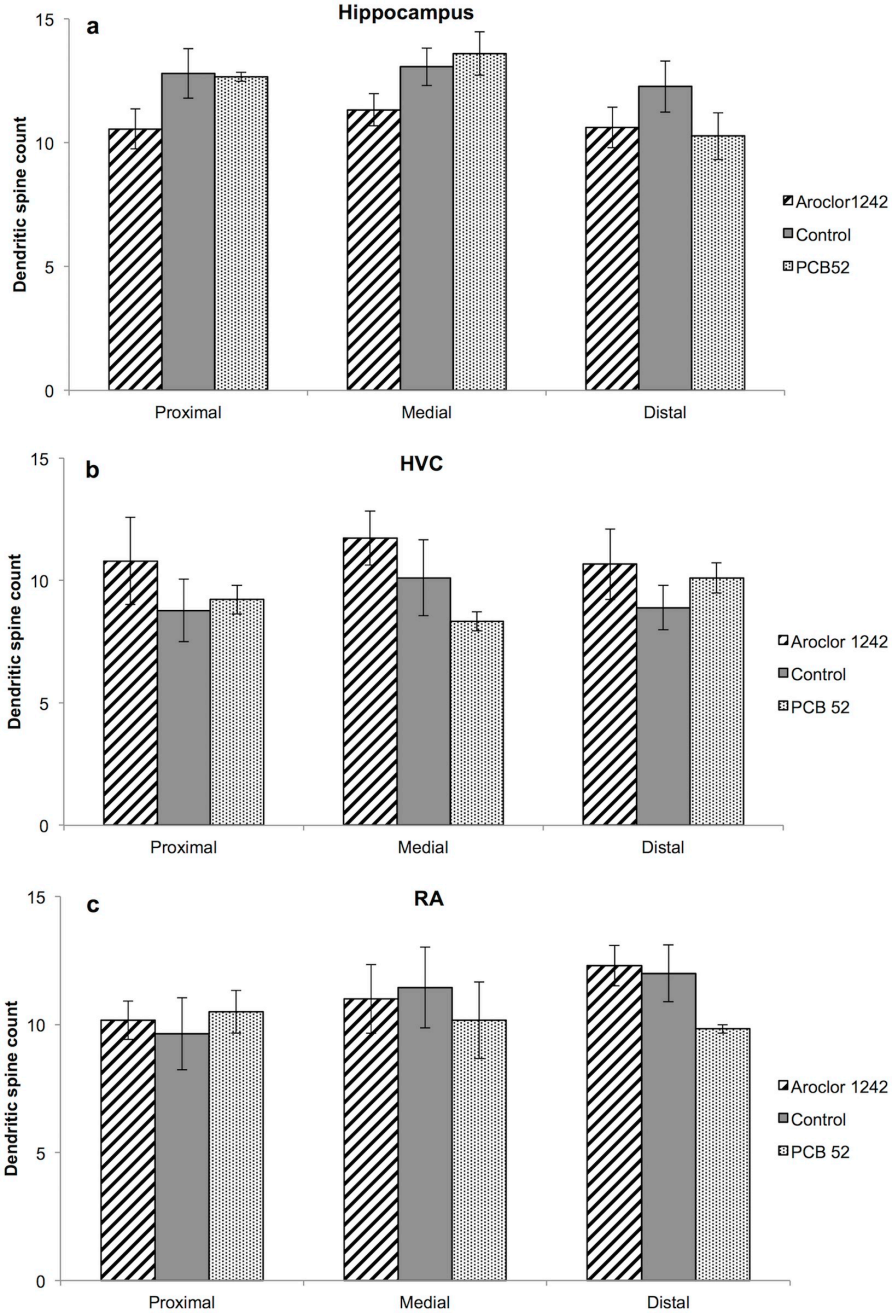
F1 male brain dendritic spine quantification. The mean value of adult brain dendritic spines in the a) hippocampus, b) HVC, and c) RA of F1 male zebra finches. Error bars indicate ±SE.

There was also no difference between treatment groups in the number of dendritic spines in the proximal, medial, or distal portions of the dendrite in the hippocampus (Student’s T-test: *t*_*13*_ = 2.16, *P>*0.05; Fig 4a), HVC (Student’s T-test: *t*_*8*_ = 2.31, *P*>0.05; Fig 4b), or RA (Student’s T-test: *t*_*11*_ = 2.20, *P*>0.05; Fig 4c).

## Discussion

Our results show that developmental exposure to sublethal PCB levels can negatively affect adult zebra finch reproductive characteristics and behaviour.

### Male song

Male zebra finches treated with PCBs in the nest sang less complex adult songs. Nestlings exposed to the pure PCB congener, PCB 52, had fewer syllables in their adult songs compared to control birds, while nestlings exposed to the PCB mixture, Aroclor 1242, did not differ significantly in any adult song characteristic we measured. Despite all subjects also being involved in the male behavioural assay, it is unlikely that this assay, per se, affected these results. Male zebra finches have crystallized adult song at the time of recording [42]. Although the environmental enrichment of the behavioural assay could have caused neural changes after the recordings [51], any morphological consequences to the brain should have equally affected all treatment groups, not changing the pattern of the results.

Our results of decreased song complexity in PCB-treated males suggest potential negative implications for wild birds. Adult female zebra finches prefer males who sing with more complex songs [52,53] and in general, mate choice studies in birds show that females of some species prefer males that sing more complex songs [54]. Accordingly, PCB exposure to nestlings may have a negative impact on their attractiveness to potential adult partners. Furthermore, in the field, male nestlings hatched in hotspots of PCB contamination may sing compromised adult songs, thereby affecting their reproductive fitness.

A limited number of studies show consequences of environmental chemical pollution on bird song, yet with divergent results. Carolina wrens (*Thryothorus ludovicianus*), house wrens (*Troglodytes aedon*), song sparrows (*Melospiza melodia*), and great tits (*Parus major*) living in areas of heavy metal pollution sing with less note diversity, fewer songs during the dawn chorus, and shorter songs compared to individuals living in less polluted environments [55,56]. Similarily, black-capped chickadees sing less stereotyped songs and song sparrows sing slower trills in areas of higher PCB pollution [32]. In contrast, European starlings (*Sturnus vulgaris*) treated with environmental estrogens sing more complex songs and female starlings preferred those songs over songs from male controls [57].

Although we found changes in adult song complexity when nestlings were treated with PCB 52, we do not see significant changes in adult song with Aroclor 1242 treatment. The different consequences of Aroclor 1242 and PCB 52 exposure is likely an effect of their different chemistry, and an effect of a mixture versus a pure PCB congener. Whereas Aroclor 1242 has an average of three chlorine atoms per molecule [58], PCB 52 is a molecule with four chlorine atoms. We suspect that the exposure to the higher chlorinated PCB 52 and the higher amount of a single non-natural estrogenic congener [59] caused mixed agonist/antagonist effects and interfered with the normal masculinization of the male song during development [10,60,61]. In contrast, the mixture of many different PCB congeners in Aroclor 1242 (many with lower chlorination) may have reduced the potency of the Aroclor 1242 treatment overall, resulting in no discernable song change in the adults.

### Male behavioural assay

Although the consequences of adult PCB-exposure on the reproductive behaviours of female zebra finches have been examined [27], to our knowledge this is the first time the effects of neonatal PCB-exposure on male birds has been the subject of experimental investigation. In our male behavioural assay, the PCB 52 treatment resulted in a shorter latency to successful clutches in comparison to the Aroclor 1242 treatment. In contrast, there were significantly more nests built before a successful nest in the Aroclor 1242 cages than in the PCB 52 cages, and females in the Aroclor 1242 cages laid significantly more eggs before a successful clutch in the Aroclor 1242 cages compared to the PCB 52 cages.

Although there were no statistical differences in observed aggressive behaviour, we suspect that aggression plays a significant role in these results. In two out of seven PCB 52 cage treatments the experiments were stopped in the pre-laying period due to heavy aggression towards the PCB 52 male from the control male, in contrast to other instances of aggression during the post-hatch period that required stopping the experiment (in the Aroclor 1242 treatment: the control male towards the Aroclor 1242 male; in the PCB 52 treatment: PCB 52 male towards the control male). While the delay to a successful first clutch in the Aroclor 1242 treatment initially appears to indicate the Aroclor 1242 cage treatment was, overall, more disruptive than the PCB 52 cage treatment, the aggressive results indicate a more complicated story. Instead it seems more likely that the highly aggressive atmosphere in the PCB 52 cages, causing the assay to be discontinued, resulted in in an overall decrease in the average observed aggression in the PCB 52 cage treatment overall, thereby facilitating a shorter latency to successful clutches. Therefore, the delay in the first successful clutch in the Aroclor 1242 cage treatment, an event important in reproductive success in a number of species (for examples see [62] and [63]), appears mainly to be a result of an absence of a dominant male in the behavioural assay.

PCB exposure has been linked to aggressive behaviour in a number of organisms. In mice, aggression was inversely proportional to PCB treatment dosage [64], while a domestic rhesus monkey (*Macaca mulatta*) breeding colony that was continuously exposed to PCBs exhibited notable trauma from other exposed members of the colony [65]. Aggressive courtship interactions were observed in captive American kestrels [24], and in humans, aggression (violence) has also been linked to early PCB exposure. Early PCB exposure in humans has been shown to lower IQ, shorten attention span, and increase the frequency of antisocial behaviour, making these individuals more likely to commit a violent crime [66,67]. Within this context it is not surprising that we observed higher levels of aggressive behaviour in the male behavioural assay.

The amount of song in the pre-laying and laying period also differed between treatments in the male bahvioural assay. During the pre-laying period control males sang more in the Aroclor 1242 cage treatment than the control males in the PCB 52 cage treatment. There was also more total singing in the Aroclor 1242 cage treatment during the laying period than the PCB 52 cage treatment. These song results further suggest a lack of dominant male and more ongoing male-male competition in the Aroclor 1242 treatment than in the PCB 52 treatment.

There was no evidence that the F1 developmental treatment affected the adult reproductive success. Moreover, because the females in the male behavioural assay were untreated and the paternity data indicated that each male F1 treatment group sired offspring, there is no indication that these PCB-treatments caused infertility. Zebra finches are a monogamous passerine [68], but are known to engage in extra pair copulations resulting in extra pair paternity in both the wild and in captivity [68,69]. Our male behavioural assay set-up put two males in direct competition with each other, likely allowing ample opportunity for frequent copulations and female guarding [70,71], and therefore explaining the low levels of mixed paternity observed in our results. However, F2 young fledged significantly earlier if they had a control genetic father than if they had an Aroclor 1242 genetic father, with F2 young with PCB 52 genetic fathers having an intermediated fledgling age. A delayed fledgling age would likely cause decreased survival in the wild, reducing reproductive success [72,73].

Disrupted reproductive behaviours due to PCB exposure have been seen in other bird species, such as the previously mentioned captive American kestrels. When exposed to a mixture of Aroclor 1248, 1254, and 1260 as adults, the effects were primarily skewed towards disrupted male kestrel behaviour and caused by a delay in clutch initiation [25]. Similarly our Aroclor 1242 treatment shows a delay of the first successful clutch in comparison to the PCB 52 treatment and a later fledgling age of the offspring with Aroclor 1242 fathers, potentially mirroring the mechanisms affected in the kestrels. In contrast, the PCB 52 treatment shows a high level of aggression early in the assay. Therefore, environmental PCB-exposure to male passerines during development may be impacting their reproductive success in the wild through consequences such as the inability to secure a mate or increased aggression. Furthermore, this study shows that sublethal neonatal exposure of male birds to PCBs may not only affect their reproductive behaviour, but also may impact the breeding success of their un-exposed, female reproductive partners.

### Female song preference

As song is vital to male zebra finch reproductive behaviour, from the females’ perspective, her mate choice decision is crucial to her reproductive success and is a strong selective force [74]. Only F1 females treated with Aroclor 1242 responded significantly to the song preference trials (Fig 3a), while control and PCB 52 treated females did not show a significant response to the trials (Fig 3). These results are difficult to interpret since the small sample of stimulus songs in these trials hinders the interpretation of the ‘attractiveness’ of the male song [75]. However, these female song preference trials do not examine which type of male song elicits a preference, but instead explores whether the expression of a preference differs between female groups. One possible explanation for the significant response from the Aroclor 1242 treated females is that the Aroclor 1242 congener mixture acts as an estrogen mimic to the F1 female zebra finches. In female zebra finches estrogen has been show to selectively heighten song responsiveness [76]. Other passerines, such as female sedge warblers (*Acrocephalus schoenobaenus*), that have been implanted with estradiol have also been shown to respond more strongly to male playback [77]. Indeed, chemicals such as PCBs are capable of disrupting aspects of mate choice, such as significantly altering the preference for a sex-specific signal (reviewed in [78]). In contrast, we suspect that in the other treatment groups male song playbacks with a stuffed male model and without the accompaniment of other cues from a live male [79] were not enough to mimic the complexity of signals needed for a clear female preference.

### Male dendritic spine quantification

In zebra finches song is learned and produced by a well-defined set of brain nuclei that are regulated, in part, by sex steroids [80,81]. The pathway of song production consists of the HVC (proper name), which projects to the robust nucleus of the arcopallium (RA), which projects to the hypoglossal (nXII) nucleus that controls the muscles of the syrinx used for vocalizations [82]. The HVC contains both androgen and estrogen receptors, while the RA contains only androgen receptors [80,83–86].

Although we found differences in adult male song characteristics, these differences were not reflected by changes to song nuclei dendritic spine count. We found no difference in the number of adult dendritic spines in the HVC or RA in either PCB 52- or Aroclor 1242- treated zebra finch nestlings. Studies on exposure to environmental chemical pollution on song nuclei in the passerine brain show that environmental estrogen increases the HVC volume in European starlings [57], while American robins (*Turdus migratorius*) from areas of DDT (1, 1, 1-trichloro-2, 2-di (4-chlorophenyl) ethane) pollution have decreased HVC and RA volumes [87]. When treated with the PCB mixture Aroclor 1248, adult female zebra finches have progeny with decreased RA volume, although there was no change to their HVC volume [88].

While we cannot conclusively say why there were no differences in the number of dendritic spines of the adult birds even though there were changes to the adult song, one possible explanation is that the time lapse between the dosage and brain analysis in our study allowed for a ‘recovery’ of the dendritic spines in our adult subjects. In the only other study we are aware of where the same bird was dosed with a pollutant and then sacrificed for song nuclei measurements in a controlled experiement, European starlings dosed as adults were then promptly sacrificed for song nuclei volume measurements after the dosage regime was finished [57]. In our experiment the perfusion and brain extraction occurred substansially after the PCB dosage regime (Fig 1). Neurogenesis is known to occur in adult passerine birds and can be influenced by circulating testosterone, singing behaviour and stress (reviewed in [89]). It is likely that all these conditions varied between the time of treatment and perfusion in our study, potentially masking any measurable neurological changes to the song nuclei.

It is important to note that although we saw no change in the number of dendritic spines in the HVC or RA, we did not measure HVC or RA volume. Although song nuclei volume is a common measurement used to determine pollutant effects on song nuclei in birds [52,73,74], due to the long time between dosage and brain analysis in our study, we did not expect to find volume changes in the HVC and RA. In the only other study we are aware of that looks at the effects of PCB exposure on zebra finch song nuclei volumes, adult laying females were exposed to Aroclor 1248 and the RA volume of day 50 progeny decreased, while there was no change to the HVC volume [88]. Furthermore, the number of dendritic spines has been successfully used to quantify neurological changes in PCB-exposed organisms [90,91]. To our knowledge, this is the first time dendritic spines have been used to investigate the effects of PCB exposure to bird song nuclei.

Additionally, we saw no changes to the number of dendritic spines in the hippocampus between our three treatment groups. Although developmental treatment with endocrine disrupting chemicals, including PCBs, have been shown to decrease spinal density in the hippocampus and alter adult behaviour in rats and mice [92–94], we saw no such results. Once again, it is possible that the time lag between the neonatal dosage and adult dendritic spine count allowed for neurogenesis to occur and masked any measurable changes to the hippocampus dendritic spines [95].

### Conclusions

PCB complexity as a chemical class necessitates that specific congeners, as well as the timing and quantity of the exposure, must all be considered when investigating potential developmental PCB-effects. The various reproductive endpoints measured in this study highlight that pure PCB congeners and PCB mixtures can have distinct consequences on biological systems. Additionally, it is evident that sublethal PCB-exposure for only a limited time during development influences both male and female adult behaviour in ways that can have significant consequences to their adult reproductive success.

In particular, we emphasize that in our treatment of sublethal PCB-levels to nestling passerines, we saw significant changes in the adult male song and reproductive behaviour, not in morphological data, brain anatomy, or traditional measure of reproductive succes. These results highlight the importance of including behavioural endpoints when investigating the effects of environmental chemical pollutants [96].

## Supporting information

S1 Fig Top view of the female song preference trial apparatus. Solid lines indicate wire aviary cage boundaries, dotted line indicates a central perch, and dashed lines with circle endings indicate movable wire-mesh gates. Stuffed male models (M) were placed directly behind and below speakers (S) at each end of the apparatus. The female was initially placed in the center of the apparatus (darker shaded area), and the time the female spent near each speaker (lighter shaded area) was recorded.
(TIF)

S1 Table F1 male and female fledging age and size. (DOCX)

S2 Table Male behavioural assay subject size. F1 male and female size at start of the male behavioural assay.
(DOCX)

S3 Table Male aggression in behavioural assay. Aggression results in the pre-laying and laying period of the male behavioural assay.
(DOCX)

S4 Table Male reproductive behaviour in behavioural asssy. Pre-laying, laying and post-hatch reproductive behavioural results of the male behavioural assay. Significant differences are indicated with an asterisk (*) and darker shaded cells.
(DOCX)

S5 Table F1 male brain and testes mass. (DOCX)
