# Supplementary figures and images for: Developmental polychlorinated biphenyl exposure influences adult zebra finch reproductive behaviour
Source: PLoS One. 2020 Mar 19;15(3):e0230283. doi: 10.1371/journal.pone.0230283 (PMC7082000; doi:10.1371/journal.pone.0230283)

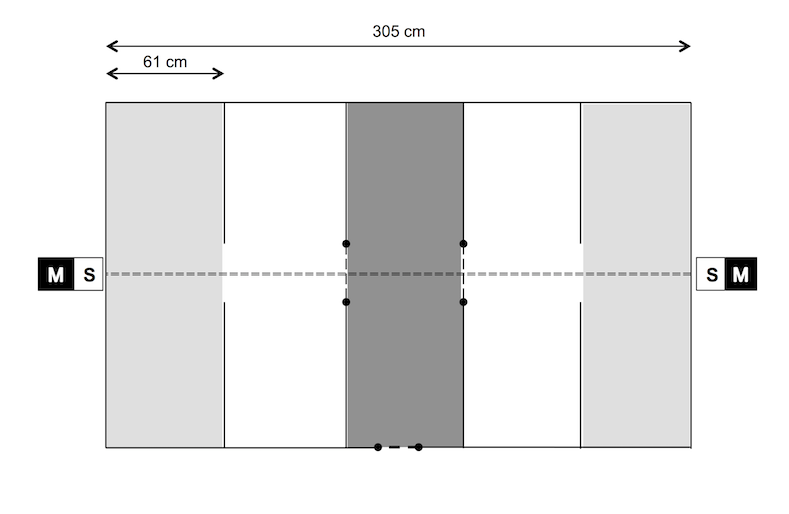

Supplement: S1 Fig — Solid lines indicate wire aviary cage boundaries, dotted line indicates a central perch, and dashed lines with circle endings indicate movable wire-mesh gates. Stuffed male models (M) were placed directly behind and below speakers (S) at each end of the apparatus. The female was initially placed in the center of the apparatus (darker shaded area), and the time the female spent near each speaker (lighter shaded area) was recorded. (TIF) [file pone.0230283.s001.tif]
