# Supplementary material for: Developmental polychlorinated biphenyl exposure influences adult zebra finch reproductive behaviour
Source: PLoS One. 2020 Mar 19;15(3):e0230283. doi: 10.1371/journal.pone.0230283 (PMC7082000; doi:10.1371/journal.pone.0230283)
Supplement: S1 Table — (DOCX) [file pone.0230283.s002.docx]

**S1 Table. F1 male and female fledging age and size.**

|  | Units | Control | Aroclor 1242 | PCB 52 | *F* | df | *P* | |
| --- | --- | --- | --- | --- | --- | --- | --- | --- |
| F1 males |  |  |  |  |  |  |  | |
| Fledgling age | days | 19.77±0.32 (*13*)^a^ | 19.71±0.36 (*7*) | 19.50±0.34 (*6*) | Kruskal-Wallis: H(3)=0.62 | | 0.74 |  |
| D20 mass | g | 12.12±0.19 (*13*) | 11.64±0.34 (*7*) | 11.58±0.30 (*6*) | 1.35 | 23 | 0.28 | |
| D120 mass | g | 13.46±0.29 (*13*) | 13.26±0.64 (*7*) | 13.17±0.44 (*6*) | 0.11 | 23 | 0.89 | |
| D20 tarsus length | mm | 14.26±0.22 (*13*) | 14.43±0.20 (*7*) | 13.75±0.13 (*6*) | 1.40 | 23 | 0.27 | |
| D120 tarsus length | mm | 14.92±0.19 (*13*) | 15.07±0.25 (*7*) | 14.51±0.20 (*6*) | 1.07 | 23 | 0.36 | |
| F1 females |  |  |  |  |  |  |  | |
| Fledgling age | days | 19.85±0.63 (*7*) | 19.00±0.26 (*6*) | 19.14±0.46 (*7*) | 0.85 | 17 | 0.45 | |
| D20 mass | g | 11.86±0.28 (*7*) | 11.33±0.25 (*6*) | 11.86±0.26 (*7*) | 0.41 | 17 | 0.67 | |
| D120 mass | g | 13.71±0.26 (*7*) | 13.75±0.31 (*6*) | 14.50±0.48 (*7*) | 0.85 | 17 | 0.45 | |
| D20 tarsus length | mm | 14.51±0.29 (*7*) | 14.23±0.13 (*6*) | 14.25±0.17 (*7*) | 0.69 | 17 | 0.52 | |
| D120 tarsus length | mm | 14.94±0.21 (*7*) | 14.25±0.15 (*6*) | 14.80±0.18 (*7*) | 1.86 | 17 | 0.20 | |

**^a^**All values are mean±SE (*N*)
