# Supplementary material for: Developmental polychlorinated biphenyl exposure influences adult zebra finch reproductive behaviour
Source: PLoS One. 2020 Mar 19;15(3):e0230283. doi: 10.1371/journal.pone.0230283 (PMC7082000; doi:10.1371/journal.pone.0230283)
Supplement: S2 Table — F1 male and female size at start of the male behavioural assay. (DOCX) [file pone.0230283.s003.docx]

**S2 Table. Male behavioural assay subject size.** F1 male and female size at start of the male behavioural assay.

|  | Units | Control | Aroclor 1242 | PCB 52 | *F* | df | *P* |
| --- | --- | --- | --- | --- | --- | --- | --- |
| F1 male mass | g | 14.04±0.31 (*13*)^a^ | 13.93±0.39 (*7*) | 13.33±0.36 (*6*) | 0.68 | 23 | 0.52 |
| F1 male tarsus length | mm | 14.31±0.21 (*13*) | 14.53±0.19 (*7*) | 14.11±0.14 (*6*) | 0.67 | 23 | 0.52 |
|  |  |  | **Aroclor 1242 cage treatment** | **PCB 52 cage treatment** | ***F*** | **df** | ***P*** |
| experimental female mass | g |  | 15.00±0.53 (*7*) | 14.17±0.49 (*6*) | 1.28 | 11 | 0.28 |
| experimental female tarsus length | mm |  | 14.29±0.25 (*7*) | 13.87±0.23 (*6*) | Kruskal-Wallis: H(2)=1.85 | | 0.17 |

**^a^**All values are mean±SE (*N*)
