# Supplementary material for: Developmental polychlorinated biphenyl exposure influences adult zebra finch reproductive behaviour
Source: PLoS One. 2020 Mar 19;15(3):e0230283. doi: 10.1371/journal.pone.0230283 (PMC7082000; doi:10.1371/journal.pone.0230283)
Supplement: S3 Table — Aggression results in the pre-laying and laying period of the male behavioural assay. (DOCX) [file pone.0230283.s004.docx]

**S3 Table. Male aggression in behavioural assay.** Aggression results in the pre-laying and laying period of the male behavioural assay.

|  | | **Control** | **Aroclor 1242** | **PCB 52** | **F-value** | **Effect size (*r^2^)*** | **P value** |
| --- | --- | --- | --- | --- | --- | --- | --- |
| *Pre-laying period* | | | | | | | |
| M/M | Aroclor 1242 treatment | 1.29±2.21 (*7*)^a^ | 1.29±1.89 (*7*) |  | Kruskal-Wallis: H(2)=0.01 | 0.00 | 0.92 |
|  | PCB 52 treatment | 1.00±1.41 (*4*) |  | 2.00±1.82 (*4*) | Kruskal-Wallis: H(2)=0.63 | 0.04 | 0.42 |
| F/M | Aroclor 1242 treatment | 0.00±0.00 (*7*) | 0.57±0.98 (*7*) |  | Kruskal-Wallis: H(2)=1.75 | 0.18 | 0.19 |
|  | PCB 52 treatment | 0.25±0.50 (*4*) |  | 0.25±0.50 (*4*) | Kruskal-Wallis: H(2)=0.13 | 0.04 | 0.72 |
| M/F | Aroclor 1242 treatment | 0.00±0.00 (*7*) | 0.14±0.38 (*7*) |  | Kruskal-Wallis: H(2)=0.00 | - | 1.00 |
|  | PCB 52 treatment | 0.00±0.00 (*4*) |  | 0.00±0.00 (*4*) | Kruskal-Wallis: H(2)=0.57 | 0.06 | 0.45 |
| *Laying period* | | | | | | | |
| M/M | Aroclor 1242 treatment | 3.33±4.46 (*6*) | 4.67±5.39 (*6*) |  | Student's T-test: t(10)=-1.53 | 0.21 | 0.18 |
|  | PCB 52 treatment | 7.33±3.21 (*3*) |  | 2.67±4.62 (*3*) | Kruskal-Wallis: H(2)=0.65 | 0.04 | 0.42 |
| F/M | Aroclor 1242 treatment | 2.67±3.33 (*6*) | 0.83±2.04 (*6*) |  | Kruskal-Wallis: H(2)=2.91 | 0.20 | 0.09 |
|  | PCB 52 treatment | 0.00±0.00 (*3*) |  | 0.00±0.00 (*3*) | Kruskal-Wallis: H(2)=0.50 | 0.06 | 0.48 |
| M/F | Aroclor 1242 treatment | 0.33±0.82 (*6*) | 0.17±0.41 (*6*) |  | Kruskal-Wallis: H(2)=0.50 | 0.06 | 0.48 |
|  | PCB 52 treatment | 0.00±0.00 (*3*) |  | 0.00±0.00 (*3*) | Kruskal-Wallis: H(2)=0.50 | 0.06 | 0.48 |

**^a^**All values are mean±SE (*N*)
