# Supplementary material for: Developmental polychlorinated biphenyl exposure influences adult zebra finch reproductive behaviour
Source: PLoS One. 2020 Mar 19;15(3):e0230283. doi: 10.1371/journal.pone.0230283 (PMC7082000; doi:10.1371/journal.pone.0230283)
Supplement: S4 Table — Pre-laying, laying and post-hatch reproductive behavioural results of the male behavioural assay. Significant differences are indicated with an asterisk (*) and darker shaded cells. (DOCX) [file pone.0230283.s005.docx]

**S4 Table. Male reproductive behaviour in behavioural asssy.** Pre-laying, laying and post-hatch reproductive behavioural results of the male behavioural assay. Significant differences are indicated with an asterisk (*) and darker shaded cells.

|  | | | | | Control | | Aroclor 1242 | PCB 52 | F-value | Effect size (*r^2^)* | P value | |
| --- | --- | --- | --- | --- | --- | --- | --- | --- | --- | --- | --- | --- |
| Pre-laying reproductive behaviour | | | | | | | | | | | |  |
| Singing | | *Within treatment* | Aroclor 1242 treatment | | | 23.43±21.10 (*7*)^a^ | 13.14±6.26 (*7*) |  | Student's T-test: t(12)=1.24 | 0.11 | 0.24 | |
|  |  |  | PCB 52 treatment | | | 4.50±4.65 (*4*) |  | 6.75±3.30 (*4*) | Kruskal-Wallis: H(2)=0.79 | 0.09 | 0.37 | |
|  |  | *Between treatment* | Total | | |  | 36.57±22.34 (*7*) | 11.25±4.57 (*4*) | Student's T-test: t(9)=-2.19 | 0.35 | 0.06 | |
|  |  |  | Control | | |  | 23.43±7.98 (*7*) | 4.50±4.65 (*4*) | Kruskal-Wallis: H(2)=4.40 | 0.25 | **0.04*** | |
|  |  |  | Aroclor 1242/PCB 52 | | |  | 13.14±6.26 (*7*) | 6.75±3.30 (*4*) | Student's T-test: t(9)=-1.87 | 0.28 | 0.09 | |
| Allopreening | | *Within treatment* | Aroclor 1242 treatment | | | 0.43±0.79 (*7*) | 1.86±3.34 (*7*) |  | Kruskal-Wallis: H(2)=0.56 | 0.09 | 0.46 | |
|  |  |  | PCB 52 treatment | | | 1.00±1.41 (*4*) |  | 0.25±0.50 (*4*) | Kruskal-Wallis: H(2)=0.69 | 0.14 | 0.40 | |
|  |  | *Between treatment* | Total | | |  | 2.29±3.30 (*7*) | 1.25±1.89 (*4*) | Kruskal-Wallis: H(2)=0.16 | 0.03 | 0.69 | |
|  |  |  | Control | | |  | 0.43±0.79 (*7*) | 1.00±1.41 (*4*) | Kruskal-Wallis: H(2)=0.59 | 0.08 | 0.44 | |
|  |  |  | Aroclor 1242/PCB 52 | | |  | 1.86±3.34 (*7*) | 0.25±0.50 (*4*) | Kruskal-Wallis: H(2)=0.59 | 0.09 | 0.44 | |
| Time in nestbox with female | | *Within treatment* | Aroclor 1242 treatment | | | 1.00±2.24 (*7*) | 0.29±0.76 (*7*) |  | Kruskal-Wallis: H(2)=0.39 | 0.05 | 0.53 | |
|  |  |  | PCB 52 treatment | | | 1.00±1.41 (*4*) |  | 0.25±0.50 (*4*) | Kruskal-Wallis: H(2)=0.69 | 0.14 | 0.40 | |
|  |  | *Between treatment* | Total | | |  | 1.28±2.21 (*7*) | 1.25±1.26 (*4*) | Kruskal-Wallis: H(2)=0.36 | <0.01 | 0.55 | |
|  |  |  | Control | | |  | 1.00±2.24 (*7*) | 1.00±1.41 (*4*) | Kruskal-Wallis: H(2)=0.30 | 0.00 | 0.58 | |
|  |  |  | Aroclor 1242/PCB 52 | | |  | 0.29±0.76 (*7*) | 0.25±0.50 (*4*) | Kruskal-Wallis: H(2)=0.08 | <0.01 | 0.78 | |
| Laying reproductive behaviour | | | | | | | | | | | |  |
| Singing | | *Within treatment* | Aroclor 1242 treatment | | | 73.00±43.42 (*6*) | 59.33±45.01 (*6*) |  | Student's T-test: t(10)=0.54 | 0.03 | 0.60 | |
|  |  |  | PCB 52 treatment | | | 25.00±9.00 (*3*) |  | 31.00±2.65 (*3*) | Student's T-test: t(4)=1.11 | 0.23 | 0.33 | |
|  |  | *Between treatment* | Total | | |  | 132.33±70.03 (*6*) | 56.00±6.56 (*3*) | Student's T-test: t(7)=-3.60 | 0.65 | **0.01*** | |
|  |  |  | Control | | |  | 73.00±43.42 (*6*) | 25.00±9.00 (*3*) | Student's T-test: t(7)=-1.83 | 0.32 | 0.11 | |
|  |  |  | Aroclor 1242/PCB 52 | | |  | 59.33±45.01 (*6*) | 31.00±2.65 (*3*) | Student's T-test: t(7)=-1.31 | 0.20 | 0.23 | |
| Allopreening | | *Within treatment* | Aroclor 1242 treatment | | | 2.83±5.23 (*6*) | 13.00±17.88 (*6*) |  | Kruskal-Wallis: H(2)=1.35 | 0.15 | 0.25 | |
|  |  |  | PCB 52 treatment | | | 1.33±2.31 (*3*) |  | 0.33±0.58 (*3*) | Kruskal-Wallis: H(2)=0.07 | 0.12 | 0.80 | |
|  |  | *Between treatment* | Total | | |  | 15.83±19.09 (*6*) | 1.67±2.89 (*3*) | Kruskal-Wallis: H(2)=1.72 | 0.18 | 0.19 | |
|  |  |  | Control | | |  | 2.83±5.23 (*6*) | 1.33±2.31 (*3*) | Kruskal-Wallis: H(2)=0.80 | 0.03 | 0.78 | |
|  |  |  | Aroclor 1242/PCB 52 | | |  | 13.00±17.88 (*6*) | 0.33±0.58 (*3*) | Kruskal-Wallis: H(2)=1.82 | 0.17 | 0.18 | |
| Time in nestbox with female | | *Within treatment* | Aroclor 1242 treatment | | | 30.83±36.72 (*6*) | 48.50±49.44 (*6*) |  | Student's T-test: t(10)=-0.70 | 0.05 | 0.50 | |
|  |  |  | PCB 52 treatment | | | 22.00±28.62 (*3*) |  | 7.67±7.51 (*3*) | Kruskal-Wallis: H(2)=0.05 | 0.15 | 0.83 | |
|  |  | *Between treatment* | Total | | |  | 79.33±59.03 (*6*) | 29.67±29.48 (*3*) | Student's T-test: t(7)=-1.34 | 0.20 | 0.14 | |
|  |  |  | Control | | |  | 30.83±36.72 (*6*) | 22.00±28.62 (*3*) | Kruskal-Wallis: H(2)=0.07 | 0.02 | 0.80 | |
|  |  |  | Aroclor 1242/PCB 52 | | |  | 48.50±49.44 (*6*) | 7.67±7.51 (*3*) | Kruskal-Wallis: H(2)=1.36 | 0.21 | 0.24 | |
| Post-hatch reproductive behaviour | | | | | | | | | | | |  |
| Singing | | *Within treatment* | Aroclor 1242 treatment | | | 45.75±18.39 (*4*) | 29.25±22.50 (*4*) |  | Student's T-test: t(6)=1.14 | 0.18 | 0.30 | |
|  |  |  | PCB 52 treatment | | | 19.00±21.21 (*2*) |  | 37.50±14.85 (*2*) | Student's t-test: t(2)=1.01 | 0.34 | 0.42 | |
|  |  | *Between treatment* | Total | | |  | 75.00±4.83 (*4*) | 56.50±36.06 (*2*) | Kruskal-Wallis: H(2)=0.06 | 0.25 | 0.81 | |
|  |  |  | Control | | |  | 45.75±18.39 (*4*) | 19.00±21.21 (*2*) | Student's T-test: t(4)=-1.61 | 0.39 | 0.18 | |
|  |  |  | Aroclor 1242/PCB 52 | | |  | 29.25±22.50 (*4*) | 37.50±14.85 (*2*) | Student's T-test: t(4)=0.46 | 0.05 | 0.67 | |
| Allopreening | | *Within treatment* | Aroclor 1242 treatment | | | 3.00±4.08 (*4*) | 0.25±0.50 (*4*) |  | Kruskal-Wallis: H(2)=2.40 | 0.23 | 0.12 | |
|  |  |  | PCB 52 treatment | | | 1.00±0.00 (*2*) |  | 2.00±2.83 (*2*) | Student's T-test: t(2)=0.50 | 0.11 | 0.67 | |
|  |  | *Between treatment* | Total | | |  | 3.25±3.95 (*4*) | 3.00±2.83 (*2*) | Student's T-test: t(4)=-0.08 | <0.01 | 0.94 | |
|  |  |  | Control | | |  | 3.00±4.08 (*4*) | 1.00±0.00 (*2*) | Kruskal-Wallis: H(2)=0.25 | 0.10 | 0.62 | |
|  |  |  | Aroclor 1242/PCB 52 | | |  | 0.25±0.50 (*4*) | 2.00±2.83 (*2*) | Kruskal-Wallis: H(2)=0.68 | 0.32 | 0.41 | |
| Time in nestbox with female | | *Within treatment* | Aroclor 1242 treatment | | | 10.50±12.15 (*4*) | 17.50±18.91 (*4*) |  | Student's T-test: t(6)=-0.62 | 0.06 | 0.56 | |
|  |  |  | PCB 52 treatment | | | 19.00±19.80 (*2*) |  | 0.00±0.00 (*2*) | Kruskal-Wallis: H(2)=2.67 | 0.48 | 0.10 | |
|  |  | *Between treatment* | Total | | |  | 28.00±24.26 (*4*) | 19.00±19.80 (*2*) | Student's T-test: t(4)=-0.49 | 0.05 | 0.67 | |
|  |  |  | Control | | |  | 10.50±12.15 (*4*) | 19.00±19.80 (*2*) | Student's T-test: t(4)=-0.56 | 0.10 | 0.65 | |
|  |  |  | Aroclor 1242/PCB 52 | | |  | 17.50±18.91 (*4*) | 0.00±0.00 (*2*) | Kruskal-Wallis: H(2)=2.18 | 0.28 | 0.14 | |
| Provisioning young |  | | Aroclor 1242 treatment | 0.25±0.50 (*4*) | | | 0.75±0.95 (*4*) |  | Kruskal-Wallis: H(2)=0.69 | 0.13 | 0.40 | |
|  |  | | PCB 52 treatment | 0.00±0.00 (*2*) | | |  | 0.00±0.00 (*2*) | Kruskal-Wallis: H(2)=0.00 | - | 1.00 | |

**^a^**All values are mean±SE (*N*)
