# Supplementary material for: Developmental polychlorinated biphenyl exposure influences adult zebra finch reproductive behaviour
Source: PLoS One. 2020 Mar 19;15(3):e0230283. doi: 10.1371/journal.pone.0230283 (PMC7082000; doi:10.1371/journal.pone.0230283)
Supplement: S5 Table — (DOCX) [file pone.0230283.s006.docx]

**S5 Table. F1 male brain and testes mass.**

|  | Control | Aroclor 1242 | PCB 52 | *F* | df | *P* |
| --- | --- | --- | --- | --- | --- | --- |
| Fresh brain mass (g) | 0.44±0.01 (*14*)^a^ | 0.43±0.01 (*6*) | 0.43±0.01 (*6*) | 0.48 | 23 | 0.63 |
| Post-Golgi-cox brain mass (g) | 0.60±0.01 (*14*) | 0.58±0.02 (*7*) | 0.58±0.02 (*6*) | 2.35 | 23 | 0.14 |
| Testes mass (g) | 0.04±0.003 (*14*) | 0.06±0.02 (*7*) | 0.04±0.004 (*7*) | 2.29 | 25 | 0.12 |

**^a^**All values are mean±SE (*N*)
